# A Manual of Human Physiology

**Published:** 1895-01

**Authors:** 


### Book Reviews.

A Manual of Human Physiology: Prepared With Special Reference to Students of Medicine. By Joseph H. Raymond, A. M., M. D., Professor of Physiology and Hygiene in the Long Island College Hospital, and Director of Physiology in the Hoagland Laboratory. With 102 Illustrations in Text and 4 Full-page Colored Plates. Philadelphia: W. B. Saunders, 925 Walnut Street. 1894.

This manual is one of "Saunders' New Aid Series," and is quite similar in many particulars to others of this class. It is intended for the use of medical students who have not time (but should have) to study a more elaborate text nor to assimilate more than the main principles and general facts of this all-important branch of medicine. The book contains about 370 pages of well-arranged, compact matter, is brief in description, impressive in language and clear to the understanding. V. H. T.

